# A Randomized Study of Rigid Video Stylet versus Macintosh Laryngoscope for Double-Lumen Endobronchial Tube Intubation Assistance in Thoracoscopic Pulmonary Surgery

**DOI:** 10.3390/jcm12020540

**Published:** 2023-01-09

**Authors:** Yang Gu, Qing Zhou, Huanping Zhou, Meiyun Liu, Di Feng, Juan Wei, Keting Min, Wanli Zhu, Yuanli Chen, Xin Lv

**Affiliations:** 1 Department of Anesthesiology, Shanghai Pulmonary Hospital, School of Medicine, Tongji University, 507, Zhengmin Road, Shanghai 200433, China; 2 Department of Anesthesiology, The Second Affiliated Hospital of Nanchang University, 1 Minde Rd, Nanchang 330006, China; 3 Graduate School, Wannan Medical College, 22 West Wenchang Rd, Wuhu 241002, China

**Keywords:** double-lumen endobronchial tube, rigid video stylet, Macintosh laryngoscope, intubation

## Abstract

Double-lumen endobronchial tube (DLT) intubation is more challenging than single-lumen tube intubation is, and the rigid video stylet (RVS) is one of the tools that has emerged to deal with this demanding intubation procedure. We evaluated whether the UE^®^ RVS can shorten the DLT intubation time and improve the first-attempt intubation success rate compared with that of Macintosh laryngoscope (ML). A total of 130 participants scheduled to undergo thoracoscopic pulmonary surgeries were enrolled. They were randomized to receive either ML- or RVS-assisted DLT intubation. The primary outcomes were the intubation time and first-attempt intubation success rate. The secondary outcomes were the overall intubation success rate, mean arterial pressure, postoperative sore throat (POST), and postoperative hoarseness at 1 h and 24 h. Compared with the ML group, the intubation time was significantly shorter in the RVS group (*p* < 0.001; 30.82 ± 10.61 vs. 39.62 ± 6.54 s), however, the first-attempt success rate was significantly lower (*p* = 0.048; 83.08% vs. 95.16%). The POST at 1 h was less severe in the RVS group (*p* = 0.021). No significant differences were found for the other indicators. Among the patients with normal airways, the UE^®^ RVS can achieve faster DLT intubation and decrease the severity of a POST at 1 h, although it was associated with a lower first-attempt intubation success rate.

## 1. Introduction

**Figure 1:**
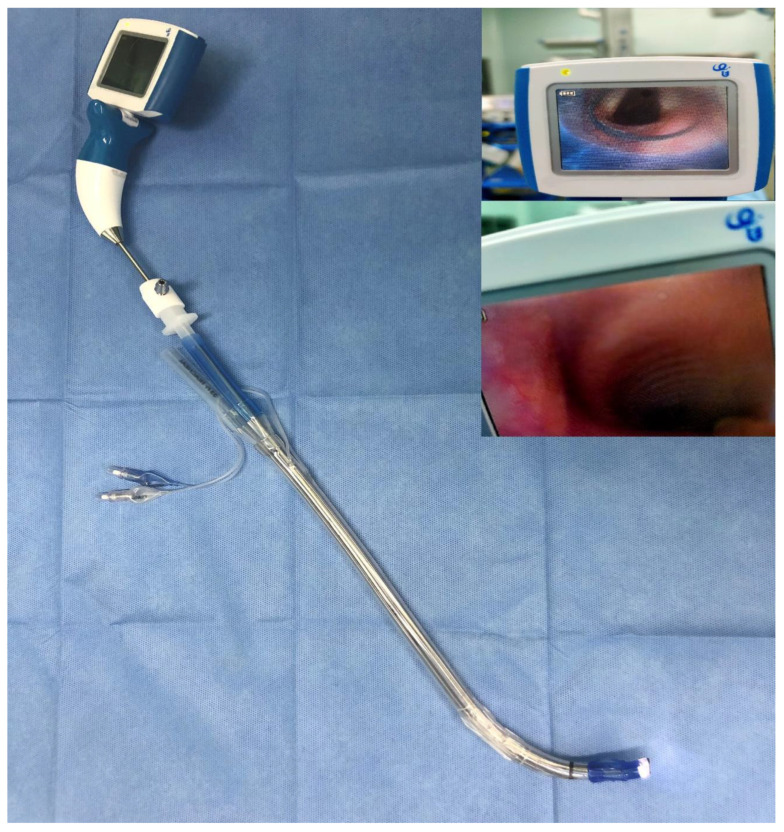
Prepared RVS with stylet in a DLT: real-time view of glottis and after endobronchial cuff passing through the glottis. Abbreviations: RVS, rigid video stylet; DLT, double-lumen endobronchial tube.

Double-lumen endobronchial tube (DLT) intubation is more challenging for anesthesiologists than single-lumen tube intubation is because DLTs are longer, larger in their outer diameter, and more rigid [1,2]. The Macintosh laryngoscope (ML) is a traditional device used in DLT intubation that is still widely used among all age groups [3]. The intubation tools have evolved significantly over the years, and the rigid video stylet (RVS) is one such emerging device. The RVS provides real-time visualization during intubation, with a stylet that is placed in the DLT. RVS-assisted endotracheal tube (ET) intubation or DLT intubation have been proven to be superior over direct laryngoscopes in terms of the intubation time in attempts on pediatric manikins or adult patients undergoing thoracic surgeries in a limited number of studies [4,5,6,7,8]. However, this method requires more experienced anesthesiologists, with the prerequisite of them having conducted more than 100 single-lumen tube and 30 DLT optical stylet-assisted intubations [5]. Furthermore, the structure of these researched video stylets and the consequences are perplexing: a short stylet requires a modification to the DLT by cutting the top to facilitate intubation, or a one-sided monitor that creates a tilting force, which is an obstacle for intubation [5,6,9]. The UE^®^ RVS (Newton, MA, USA) has a monitor in the center connected to a non-bendable stylet which is long enough (a length of 400 mm, a diameter of 5.2 mm, and an angle of 80°) to avoid need for the modification and tilting force (Figure 1). We hypothesized that this improved RVS (UE^®^) can reduce the intubation time and increase the first-attempt intubation success rate compared with ML-assisted DLT intubation. We also aimed to compare the overall intubation success rate and the early and late intubation influences on the patients.

## 2. Materials and Methods

This prospective, parallel, randomized study was approved by the Research Ethics Committee of our hospital (K19-003) and registered for clinical trials (ChiCTR1900021680), and conducted in a single center tertiary specialized hospital. Informed written consent was obtained from each participant prior to their enrollment. This study was conducted from March 2019 to December 2019.

The adult patients recruited in this study were scheduled to have a lobectomy, a segmentectomy, or a wedge resection of either of their lungs under thoracoscopy and lung isolation with left-sided DLTs. Individuals with symptomatic chronic pharyngitis, a sore throat or hoarseness, suspected difficult airways (body mass index > 30; modified Mallampati class III, IV; other inferences lead to difficult airway by the evaluator), pneumectomy, or left-sided sleeve resection were excluded. This exclusion decision was executed by a blinded anesthesiologist during the preoperative evaluation who also asked for permission from the patient and the family for this trial inclusion. The drop-out criteria were intubation being achieved by a video laryngoscope, delayed extubation (over 30 min after the termination of the surgery), delirium or postoperative cognitive dysfunction, post-operative emergent re-operation in the following 24h, perioperative intravenous dexamethasone that may ameliorate postoperative sore throat (POST) or postoperative hoarseness (PH) [10], perioperative major cardio-thoracic or cerebral events, and the patients’ refusal to attend the follow-ups at any time.

A statistician achieved randomization using a computer-generated random number table (https://www.randomizer.org/), and then, they allocated those numbers equally to group 1 (ML group) or group 2 (RVS group) patients in opaque envelopes. Thereafter, the statistician was no longer engaged in the study to preserve this allocation concealment. The opaque sealed envelope containing the directions for the intubation group was revealed to the anesthesiologist before the DLT preparation (Shiley^®^, Covidien, MA, USA. 35/32 Fr for women; 39 Fr for men), and the patients were sedated by a senior staff member. The preoperative evaluation and recruitment were performed by an anesthesiologist who was not involved in subsequent clinical anesthesia and postoperative evaluations. All of the participating patients and the staff members involved in the follow-ups and intraoperative evaluations were blinded to the group assignments, except for the intubating anesthesiologists and the research nurse. This blinding continued until the data analysis was completed. The recruitment lasted until there were patients enough in both of the groups.

Radial artery cannulation for continuous hemodynamic monitoring was performed after standard monitoring and sedation in the operating room; the mean arterial blood pressure (MAP) was recorded. Rapid sequence induction was carried out by the same senior staff member after 3 min of preoxygenation with midazolam (0.03 mg/kg), sufentanil (0.3–0.6 μg/kg), propofol (1–1.5 mg/kg), and rocuronium (0.6–0.8 mg/kg) injections. This senior staff also assisted with the subsequent intubations and evaluated the intubation success rate. Sixty seconds after the rocuronium injection, the intubation was performed by an anesthesiologist with more than 8 years of clinical anesthesia experience and over 50 DLT intubations assisted by the RVS before the start of this trial.

In the ML group, the DLT was turned 90° counterclockwise after the stylet was withdrawn by the senior staff and the endobronchial cuff was inserted through the glottis. Then, the DLT was advanced downward until they felt resistance; the insertion depth was noted. In the RVS group, the prepared DLT was inserted after the mouth was opened using the left hand. During the insertion, the left hand adjusted the mandible and tongue so that plausible space could be created for DLT advancement. During advancement, the operator watched on the monitor until the endobronchial cuff passed through the glottis, which is when the senior staff was asked to withdraw the RVS (Figure 1). Then, the DLT was turned 90° counterclockwise and advanced downward until they felt resistance. In both of the groups, the senior staff member assisted with the BURP maneuver (backward, upward, and right-sided pressure) on the thyroid and cricoid cartilages. The patients were placed in a supine position without a pillow, and intubation without clear sight of the glottis was allowed in both of the groups. A bronchofiberscope was utilized for positioning if it was necessary.

The research nurse recorded the intubation time, which began with the mouth opening maneuver and ended with DLT advancement discontinuation. Intubation success was based on the end-tidal CO_2_ on the monitor during manual ventilation by the senior staff. Each attempted intubation time was limited to 120 s, and if it was not achieved within this limit, intubation would be stopped by the senior staff, and mask ventilation was applied to secure oxygenation. Another attempt was allowed with the same time limit, however, if the second attempt failed, mask ventilation was reapplied. Meanwhile, the senior staff prepared a new DLT for intubation assisted by a video laryngoscope, which was recorded as a failed intubation. The intubation time was processed as missing data if an intubation failure occurred. The research nurse also recorded the MAP after induction (MAP1) and at 1 min after the successful intubation and fixation (MAP2). Before the patients were placed in the lateral position, another blinded anesthesiologist evaluated the injuries to the lips, teeth, and oral cavity caused by the intubation.

One-lung ventilation anesthesia maintenance was carried out with a pumped infusion of propofol and remifentanil, intermittent injections of rocuronium and sufentanil, and SpO2 maintenance at 95–100%, with the fraction of inspired oxygen being 0.5–1.0, as suggested by Karzai et al. [11]. The patients were extubated after a comprehensive evaluation of respiration and consciousness in the operating room, and then, they were transferred to the post-anesthesia care unit (PACU). Patient-controlled intravenous analgesia was administered to all of the patients.

One hour later, when the patient was completely awake in the PACU, a third blinded anesthesiologist was asked to evaluate the POST and PH. POST was based on a numeric rating scale, which was scored from 0–10, with 0, 1–3, 4–6, and 7–10 representing no sore throat, a mildly sore throat, a moderately sore throat, and severely sore throat, respectively [12,13]. Hoarseness was classified into four levels: 0, no hoarseness at all; 1, the patient complains of hoarseness, although it was not observed by the evaluator; 2, symptomatic hoarseness; 3, a loss of voice [6]. Another blinded anesthesiologist evaluated the POST and PH on the next day. If the patients were not eligible, as mentioned above, for the follow-ups, the POST and PH data were processed as missing data.

### 2.1. Outcome Variables

The primary outcomes were the intubation time from the mouth opening maneuver to the DLT advancement discontinuation during the first or second attempt; the first-attempt intubation success rate was defined as the first successful intubation for each patient in both of the groups. Regarding the secondary outcomes, we assessed the overall intubation success rate ((first attempt success + second attempt success)/patient number in each group), delta MAP (MAP2-MAP1), blood and saliva evaluations, and the POST and PH at 1 h and 24 h.

### 2.2. Sample Size

The intubation time was one of the primary outcomes. Therefore, we conducted a pilot study on intubation time in ML-assisted DLT intubation in 15 patients (mean ± standard deviation (SD), 39 ± 9.36 s). We expected the intubation time to be more than 5 s shorter in the RVS group, with a 15% dropout rate. The sample size calculation was performed using PASS software (Power and Sample Size Calculation, version 11.0.7, NCSS, LLC. Kaysville, UT, USA), which indicated that a total of 130 patients were required, with a *p*-value of < 0.05, 80% statistical power and type-I error rate 5%.

### 2.3. Statistical Analysis

Statistical analyses were performed using SPSS version 22.0 software for Windows (SPSS Inc.; Chicago, IL, USA). Data are expressed as the mean ± SD, median and number, or frequency and proportion. The significance level was set at 0.05 for each hypothesis. Missing data on the intubation time (originating from intubation failure), POST, and PH (lost to follow-ups) were recorded, but they were excluded from the significance analysis between the two groups. The data distributions were analyzed using the Kolmogorov–Smirnov test or Skewness and Kurtosis tests. For the normally distributed data, the intubation time was analyzed using 2 independent sample *t*-test. For the non-normally distributed data (delta MAP), Mann–Whitney U test was applied. Levene’s test was used for the equality of variances. We used the Wilcoxon rank-sum test to analyze the ranked data for POST and PH, while χ^2^ test (continuity correction if 1 < frequency < 5; Fisher’s exact test if frequency < 1) was used to evaluate the first-attempt intubation and overall success rates between the two groups.

## 3. Results

### 3.1. Demographic and Perioperative Data

**Figure 2:**
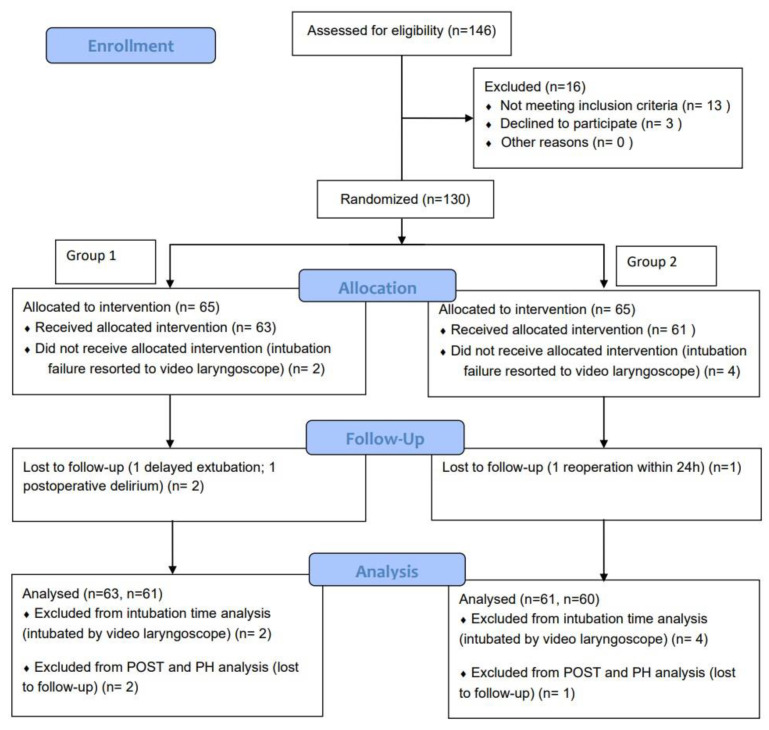
CONSORT diagram of this trial. Abbreviations: CONSORT, Consolidated Standards of Reporting Trials; POST, postoperative sore throat; PH, postoperative hoarseness.

Of the 146 patients who were evaluated for eligibility, 130 of them were included for randomization, with 65 of them being in each group. The intended intubation methods failed in two patients in the ML group and four patients in the RVS group, thus, intubation times were analyzed in sixty-three patients and sixty-one patients, correspondingly. The first-attempt intubation success rates were assessed in all of the recruited patients. A total of 61 patients in the ML group and 60 patients in the RVS group were analyzed for the POST and PH because one of them had delayed extubation and another one had postoperative delirium in the ML group who was lost to follow-ups and one patient required emergent reoperation within 24 h after the surgery in the RVS group (Figure 2). The two groups were comparable in terms of sex, age, height, weight, operation side, DLT duration, and American Society of Anesthesiologists (ASA) physical status (Table 1).

### 3.2. Intubation Time and Success Rate

The intubation time was significantly shorter in the RVS group (n = 61) than it was in the ML group (n = 63; *p* < 0.001; 30.82 ± 10.61 vs. 39.62 ± 6.54 s; mean difference (95% confidence interval), 8.80 (5.65–11.95)). However, the first-attempt intubation success rate was significantly lower in the RVS group (*p* = 0.048; 83.08% vs. 95.16%). Seven out of eleven intubation attempts were successful in the RVS group, while only one out three of them in the ML group was successful. No significant difference was found between groups in the overall success rate (*p* = 0.676; 93.85% vs. 96.92%, respectively). (Table 2).

### 3.3. Intubation Influences

The influence of intubation is presented in Table 3. The delta MAP was not significantly different between the two groups (*p* = 0.707). The intubation injury evaluations of blood and saliva were not well implemented, and they were abandoned because the group assignments were easily discerned by the evaluator. The POST at 1 h was significantly more severe in the ML group (*p* = 0.021; 95% CI, (0.016, 0.021)). The amount of alleviation was considerable in both of the groups, and no significant differences were found during the next follow-up period (*p* = 0.077). Regarding the PH, no significant differences were detected at either of the follow-ups (1 h, *p* = 0.660; 24 h, *p* = 0.376).

## 4. Discussion

In this study, we demonstrated that RVS-assisted left-sided DLT intubation can be faster, however, the first-attempt success rate was lower in comparison to the ML. The POST at 1 h was less severe in the RVS group. The overall success rate, delta MAP, POST at 24 h, and PH were not significantly different between the two groups.

Video laryngoscope-assisted DLT intubation has been proved to be superior over traditional direct laryngoscope in terms of the intubation time and glottic view in multiple studies [14]. Not only the assisting tools that have been developed but DLT itself could be equipped with a video system, and this consequently reduces the need for fiberoptic bronchoscopy [15], with which the intubation time could be shortened and the success rate could be improved for senior anesthesiologists in a manikin model [16]. Limited studies on video stylets in ET/DLT intubations have demonstrated their values in clinical use. Hsu et al. [6] proved that the Trachway^®^ stylet could achieve faster left-sided DLT intubation (24 ± 4 s) compared with that of ML (48 ± 11 s) in a total sample size of 60. In a clinical study with a much larger sample size, Yang et al. [5] found that compared with ML, the Optiscope^®^ RVS (Pacific Medical, Seoul, Republic of Korea) not only reduced DLT intubation time (15 s [12,13,14,15,16,17,18,19], but it also improved first-attempt success rates significantly in both left- and right-sided DLT intubation. Seo H and colleagues [17] even successfully used Clarus stylet-assisted DLT intubation for a patient with a possibly difficult airway. As mentioned above, the newly developed UE^®^ RVS in our study has a video monitor in the center and an adjustable stylet length for the DLT, thereby avoiding the tilting force that induces an intubation obstacle [5], as well as the need for the DLT modification [6]. Our data on intubation times are consistent with those in the aforementioned studies. The intubation time definition in those studies, however, did not start until DLT was put in the mouth and stopped after the removal of the stylet. In contrast, we recorded this as the time from just before the mouth opening maneuver was performed to the discontinuation of the DLT advancement, which may explain the longer intubation time in our study (30.82 ± 10.61 s). Moreover, we initiated intubation 60 s after the 0.6–0.8 mg/kg rocuronium injection, although it is possible that this dosage was not enough to create a perfect muscle relaxation for intubation [18]. The limited oropharyngeal space may explain the easier intubation with the RVS, considering the additional space needed for the blade in the ML group. The shorter that the intubation time spent in apneic patients was, the less likely it was the hypoxemia could occur. In contrast to a previous study [5], which set the upper limit of intubation time at 180 s and three intubation attempts, we set the upper limit at 120 s and two attempts, however, no intubations exceeded 90 s, and therefore, no patients experienced an oxygen saturation level of <95%. We may have failed to achieve more successful intubations because we prioritized the patients’ safety, however, balancing the risks versus benefits to the patients was of utmost importance.

Aside from the intubation time, the intubation success rate, especially the first-attempt success rate, is crucial in emergent airway management when adverse events are considered [19]. In contrast to previous studies on video stylet-assisted DLT intubations [4,5,6], our results showed significantly lower first-attempt success rates. Secretions, fogging caused by the temperature difference between the cold RVS lens and warm body, and a red blush appearing due to bleeding during insertion all contributed to obscured visibility, which required a second intubation to be attempted, which was avoided in the ML group. Certain precautions such as intravenous glycopyrrolate, preheating the lens in warm saline before intubations [20], and a gentle approach may lead to higher first-attempt success rates. The patients we recruited were supposed to have normal airways, however, preoperative airway evaluation screening tests have relatively low sensitivities and high variability [21]. The tongue size and teeth status also played important roles in shaping the degree of intubation difficulty. A larger tongue entails a harder pulling strength to pave the passage for visibility and DLT advancement, which would be exacerbated with a drooping tongue if neuromuscular relaxants are used. In the RVS group, creating this passage relied on the strength and technique of the operator’s fingers and assistance from senior staff; the operator had additional concerns if the patient’s lower incisors were in poor condition. The ML group differed since the blade is longer than the fingers and more flexible in manipulating the tongue, contributing to a better operating experience. Since the two intubation devices are not ideal for difficult airways, even with the help from the senior staff with the BURP or OELM (optimal external laryngeal manipulation) maneuver [22], we did not succeed in all of the intubations in either of the groups. The first-attempt success rate may have been better if the jaw thrust maneuver had been performed by a senior staff [23,24] and if we had adopted the precautions we mentioned. As we can see, most of those failed first attempts were in the RVS group, but they succeeded in the second attempt (7/11, 63.64%), contributing to a comparable overall success rate in the ML group. The operator’s experience in knowing what to expect and how to react was crucial to success in the second try.

It taking less time and there being fewer failures and injuries are three important aims in all intubation procedures. We used delta MAP to evaluate the instant influence of intubation. This indicator eliminated the induction effects, and it seemed to be more reasonable for evaluating the intubation effect itself. Similar to previous studies [5,6], no significant difference was detected between the two groups. The later intubation influence indicators we chose were POST and PH. We can infer from our results that the uplifting maneuver or the advancement of the blade used in the ML group was more traumatic, although the injuries were temporary and self-healing. Various factors in intubated patients can result in a POST, including tube size, cuff pressure, female sex, prolonged duration of surgery, or even smoking history [25,26], aside from the intubation procedure itself. Most of the patients in our study who experienced a POST in the PACU (n = 111) reported completed relief (n = 91) during the second evaluation. Regarding PH, moderate evidence [27] indicated that video laryngoscope-assisted intubation results in significantly less hoarseness than it did with the ML. Similar studies on RVS-assisted DLT intubation compared with those with the ML are very limited; no significant difference was found in our study, whereas Hsu et al. [6] found more hoarseness with ML-assisted DLT intubation. When PH is induced by some factors such as laryngeal trauma, hematoma, and edema after intubation, induction without neuromuscular blocking agents and inexperienced anesthesiologists, it could be easy to recover [28,29]. However, when trauma-induced PH is related to arytenoid cartilage dislocation or recurrent laryngeal nerve injury, the patient would require a longer recovery period [30].

### Limitations of the Study

This study had some limitations. First, the recruited subjects could not well represent patients without difficult airways, as patients were excluded for having a suspected difficult airway based on two preoperative evaluations (body mass index > 30 and modified Mallampati class III–IV). Therefore, patients with difficult airways may have been included [31]. Meanwhile, the RVS may be favorable in some patients with difficult airways, such as edentulous patients, patients with small mouth openings, or with restricted cervical spine motion [32]. Second, the intubation process was sometimes completed with the assistance of senior staff that was not recorded, which influenced both intubation times and success rates. Third, the senior staff member’s awareness of the groups and their intervention was of utmost importance in guaranteeing patient safety and reducing unnecessary harm, however, it introduced an observer bias.

## 5. Conclusions

Overall, our study suggests that in elective patients, UE^®^ RVS-assisted DLT intubation by experienced anesthesiologists can be faster, although a lower first-attempt success rate should be noted. Further studies concentrating on RVS application in special patients with limited mouth openings and in edentulous patients may also be of interest.

## Figures and Tables

**Table 1:** Demographic and perioperative data.

Parameter	ML (n = 65)	RVS (n = 65)
Sex (M/F)	31/34	28/37
Age (yr)	58.78 ± 9.52	57.23 ± 11.18
Height (cm)	165 ± 7.0	164 ± 7.4
Weight (kg)	66.2 ± 11.10	64.38 ± 10.78
Modified Mallampati class(I/II)	42/23	47/18
Side of the operation (Left/Right)	26/39	31/34
DLT duration (min)	93.40 ± 24.83	90.20 ± 27.89
ASA physical status (I/II/III/IV)	20/36/9/0	24/34/7/0

Data are expressed as mean ± SD or numbers. Abbreviations: ML, Macintosh laryngoscope; RVS, rigid video stylet; DLT, double-lumen endobronchial tube; ASA, American Society of Anesthesiologists.

**Table 2:** Intubation time and success rate.

Variables	ML (n = 65)	RVS (n = 65)	Mean Difference (95%CI)	*p* Value
Intubation time(s)	39.62 ± 6.54 (n = 63)	30.82 ± 10.61 (n = 61)	8.80 (5.65–11.95)	<0.001
First attempt intubation success rate	62 (95.16%)	54 (83.08%)	(2.10–2.70%)	0.048
Overall intubation success rate	63 (96.92%)	61 (93.85%)	(42.50–44.50%)	0.676

Data are expressed as mean ± SD or frequency (proportion). Missing data were not analyzed. Abbreviations: ML, Macintosh laryngoscope; RVS, rigid video stylet; CI, confidence interval.

**Table 3:** Instant and later intubation influences.

Variables	ML (n = 65)	RVS (n = 65)	95%CI	*p* Value
Delta MAP	10 (n = 65)	10 (n = 65)	(0.699–0.717)	0.707
POST 1 h (no/mild/medium/severe)	1 1/35/23/2	1 9/33/15/3	(0.016–0.021)	0.021
POST 24 h (no/mild/medium/severe)	0 42/19/0/0	0 49/10/1/0	(0.085–0.096)	0.077
PH 1 h (0/1/2/3)	1 29/15/13/4	0 31/11/13/5	(0.655–0.673)	0.660
PH 24 h (0/1/2/3)	0 58/0/3/0	0 55/1/1/3	(0.303–0.321)	0.376

Data are expressed as median (numbers/frequency). Missing data were not analyzed. Abbreviations: ML, Macintosh laryngoscope; RVS, rigid video stylet; CI, confidence interval; MAP, mean arterial pressure; POST, postoperative sore throat; PH, postoperative hoarseness.

## Data Availability

The datasets used and/or analyzed during the current study are available from the corresponding author on reasonable request.
